# Effects of acupuncture to decrease adverse events in patients with high-risk non-muscle invasive bladder cancer receiving induction intravesical BCG therapy: Study protocol for a randomized, controlled pilot and feasibility study^☆^

**DOI:** 10.1016/j.conctc.2022.101044

**Published:** 2022-12-05

**Authors:** Sarah P. Psutka, Susan Veleber, Jonathan Siman, Samia Jannat, Sarah Holt, Jonathan L. Wright, Heather Greenlee

**Affiliations:** a Department of Urology, University of Washington School of Medicine, United States; b Integrative Medicine Program, Division of Supportive Care, Fred Hutchinson Cancer Center, United States; c Division of Public Health Sciences, Fred Hutchinson Cancer Center, United States; d Clinical Research Division, Fred Hutchinson Cancer Center, United States; e Division of Medical Oncology, Department of Medicine, University of Washington School of Medicine, United States

**Keywords:** Intravesical bacillus calmette-guerin, BCG, High-risk nonmuscle-invasive bladder cancer, Acupuncture

## Abstract

**Background:**

Treatment-related serious adverse events (SAEs) are common in patients receiving intravesical Bacillus Calmette-Guerin (BCG) for the treatment of high-risk nonmuscle-invasive bladder cancer (NMIBC). Here we describe the protocol of a randomized, attention/waitlist-controlled feasibility pilot study testing the use of acupuncture to decrease SAEs and treatment interruptions in this population. The primary objectives are to evaluate the feasibility and efficacy of conducting pre-procedure acupuncture in a Urology Clinic.

**Methods:**

A total of 45 patients will be recruited and randomized in a 2:1 ratio (treatment arm: attention/waitlist control). Eligibility criteria include 1) age 18 years or older, 2) English-speaking, 3) high-risk NMIBC, 4) no acupuncture in the previous 3 months, and 5) willing and able to participate in trial activities. Patients in the treatment arm will receive acupuncture prior to weekly BCG for a total of six weeks. Methods were developed to train and monitor research acupuncturists and included online and in-person training, study manuals, and monthly meetings throughout the study period. Feasibility assessments include evaluation of the recruitment, retention and protocol adherence to acupuncture treatment, and measurement of CTCAE v5.0 adverse events specific to acupuncture, and clinic staff surveys regarding the intervention impact on clinic workflow. Efficacy measures will be compared between treatment and control groups including: EORTC-QLQ-NMIBC-24, EORTC-QLQ-C30, CTCAE v5.0, medication journal, healthcare utilization, current use of complementary, alternative, and integrative therapies, and acupuncture expectancy and treatment preference. Trial results will inform the design of a multi-center trial to expand testing of the protocol to a larger patient cohort.

## 1 Introduction

In 2021, the American Cancer Society estimates that approximately 83,730 Americans will receive new diagnoses of bladder cancer, of which approximately 70% are nonmuscle-invasive bladder cancer (NMIBC) [1]. As per the American Urological Association (AUA) Guidelines, the cornerstone of management of high-risk NMIBC is an induction intravesical Bacillus Calmette–Guerin (BCG) treatment, administered as 6-weekly instillations, starting two to six weeks following transurethral resection of the bladder tumor [2]. Unfortunately, nearly 70% of patients receiving BCG report severe adverse events [3] and approximately 17% of patients receiving induction BCG therapy necessitate treatment interruptions secondary to local or systemic adverse events including pelvic pain, dysuria, severe urgency, frequency, urge incontinence, nocturia, and/or infectious complications [4]. These treatment-associated symptoms are problematic, resulting in decreased ability to adhere to the full course of treatment, which is associated with decreased clinical efficacy of BCG in reducing the risk of recurrence and progression of bladder cancer, as well as severely negatively impacting patients’ quality of life. Consequently, there is a need for adjunctive therapies to reduce BCG-associated side effects in order to maximize adherence to the full course of therapy.

Many of the side effects associated with induction intravesical BCG mirror those of overactive bladder syndrome (OAB). Commonly, the irritative voiding complaints are managed symptomatically with anticholinergic medications, while bladder pain and dysuria are treated with local analgesics, antibiotics [5], nonnarcotic pain medications, and/or narcotics, which are variably successful in mitigating BCG-associated SAEs [5], albeit with often dose-limiting side effects.

Recently, acupuncture has been shown to effectively reduce the severity of OAB symptoms, resulting in decreased medication use and decreased need for surgical treatment of OAB, with an excellent safety and tolerability profile [[6], [7], [8], [9], [10], [11]]. Data from animal studies suggest several physiologic mechanisms of action of the beneficial effects of acupuncture on overactive bladder (OAB) syndrome, including modulating the voiding reflex by decreasing intravesical (bladder) pressure and facilitating storage through inhibiting bladder muscle motor activity [6,9,12]. Human trials in OAB show that acupuncture is associated with improvements in urgency, frequency, nocturia, incontinence, quality of life and some objectively measured improvements in urodynamic testing [[6], [7], [8]]. Additionally, acupuncture is comparable to anticholinergic drugs in the reduction of micturition episodes over 24 h, resulting in increased voided volumes and reduction in OAB symptom score with fewer reported side-effects [[6], [7], [8]]. Percutaneous Tibial Nerve Stimulation (PTNS), an established FDA-approved second-line treatment option for refractory OAB, is derived from acupuncture theory and techniques [13]. Given this preclinical data and data supporting the use of acupuncture in patients with OAB, we hypothesize that acupuncture may be a viable approach to decrease irritative bladder symptoms caused by intravesical BCG instillation in patients with NMIBC.

To our knowledge, to date, there are no specific data evaluating the efficacy of acupuncture with respect to improving treatment adherence to or mitigating side effects of induction intravesical BCG in patients with NMIBC. Here we describe the protocol of a randomized, attention/waitlist-controlled pilot and feasibility study testing the effects of an acupuncture protocol on reducing SAEs and treatment interruptions in patients receiving BCG therapy for the treatment of NMIBC.

## 2 Methods

### 2.1 Trial design overview

**Fig. 1:**
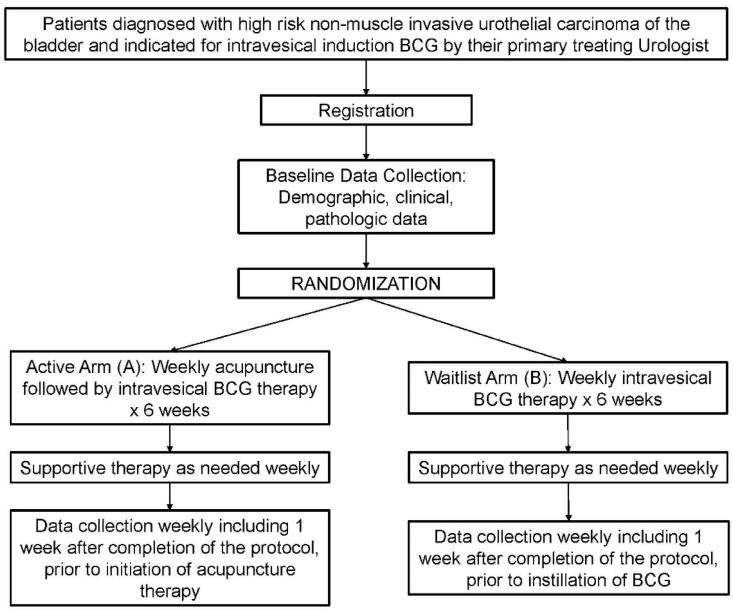
Study schema.

The trial is an 8-week, two-arm trial using a 2:1 ratio for acupuncture and attention/waitlist control, respectively (Fig. 1). Patients were randomized 2:1 (Active:Attention/Waitlist Control) and assigned using a random permuted block design. The target recruitment will be forty-five patients. Patients in the acupuncture arm receive acupuncture once weekly for 6 weeks (*i.e.*, 6 sessions) while patients in the attention/waitlist control receive standard of care BCG instillations without additional preprocedural acupuncture. Attention/waitlist control is a method used to minimize differences in experience between the acupuncture intervention and attention/waitlist control arms. Patients in the attention/waitlist control arm will be escorted to the same Consultation room where the acupuncture is performed and asked to lie in a comfortable position (similar to Arm A) prior to their intravesical therapy for an identical period of time before being moved to a treatment room for intravesical instillation of BCG. As an incentive for trial participation, patients randomized to the attention/waitlist control arm will receive vouchers at the follow-up-assessment visits after completion of the study for four true acupuncture “bonus” sessions to be used within three months.

### 2.2 Study objectives

The objective of this proposal is to evaluate the feasibility of 6-weekly pre-procedural acupuncture treatments compared to an attention/waitlist control. Secondary objectives include assessment of pre-procedural acupuncture on patient-reported bladder symptoms and overall quality of life, treatment adherence, and safety and tolerability among patients receiving intravesical BCG for NMIBC.

The feasibility of integrating outpatient acupuncture treatments into a Urology clinic workflow will be assessed by evaluation of trial recruitment, retention, and protocol adherence. Specifically, the feasibility outcome will be assessed via the following measures: 1) comparison of proportion of patients successfully recruited to the trial from those eligible, 2) assessment of the proportion of recruited patients successfully retained in the experimental (acupuncture) and control arms, 3) rates of adherence to the protocol (completion of acupuncture treatments in the experimental arm), Regarding implementation assessment; clinic staff will be surveyed regarding the impact of this intervention on clinical workflow, delays, and clinic burden. To do so, we will obtain a qualitative description of the clinic staff's responses to surveys assessing the healthcare burden of this protocol and compare timing of patient's experience within the clinic, time spent undergoing the acupuncture therapy for the experimental arm.

Finally, we will evaluate safety by tracking CTCAE v5.0 adverse events associated with acupuncture therapy in the experimental arm as completed by a provider administered standardized symptom form across weeks of therapy and describe any complications or adverse effects of the therapy.

The secondary objectives of the study are to test whether acupuncture may reduce BCG-associated side effects, and improve rates of completion of the induction BCG protocol (e.g. 6 weekly treatments). To achieve this objective, rates of 1) BCG treatment adherence between arms, as measured by treatment completion, treatment interruptions, and tolerance of treatment retention as measured by time retained, and dose reduction and 2) patient-reported outcomes of urinary symptoms and overall quality of life between arms, as measured by the EORTC QLQ-NMIBC-24 [14] and EORTC QLQ-C30 [15] scales, respectively were compared. The use of medications for symptom management in patients in the intervention and attention/waitlist control arms, as measured by medication use questionnaire, will be also quantified and compared.

### 2.3 Eligibility criteria, recruitment, and consent

This trial will recruit 45 adult patients with NMIBCs who are scheduled to receive 6 weekly doses of intravesical BCG at the UWMC Department of Urology. Patients will be indicated to receive BCG by their primary treating urologist following evaluation of histology from bladder biopsy and/or transurethral resection of bladder tumor tissue, as part of guideline-based standard of care [16].

Eligibility criteria include: 1) age 18 years or older, 2) English-speaking, 3) diagnosis of American Urological Association (AUA) high-risk NMIBC, including high grade (HG) pT1 (invading only the lamina propria of the bladder), recurrent HG pTa (non-invasive tumors), HG pTa >3 cm in size or multifocal, any carcinoma in situ, any variant histology, any lymphovascular invasion, and HG prostatic urethral involvement [17], 4) diagnosis with urothelial carcinoma (primary histologic subtype), localized to the bladder, in the absence of nodal or other visceral metastases 5) no prior acupuncture in the previous 3 months, 6) patients who have been indicated for induction intravesical BCG in shared-decision-making with their primary urologist, 7) access to phone for study contacts, 8) willing and able to participate in trial activities, 9) platelets: 20,000/μL or greater, 10) ANC: 500 cells/μL or greater, 12) ability to understand and the willingness to sign a written informed consent document, and 12) able to understand and willing to sign written informed consent in English.

Exclusion criteria include: 1) subjects who have had intravesical or systemic chemotherapy or radiation therapy for bladder cancer or for other malignancies prior to entering the study, 2) receipt of other investigational agents or other combined intravesical therapies. 3) muscle-invasive bladder cancer, radiographic evidence of lymph node metastases or metastatic disease involving other organs including brain metastases. 4) predominant histology other than urothelial carcinoma of the bladder who would not otherwise be considered candidates for BCG, 5) patients with contraindications to BCG including pregnancy, lactation, active tuberculosis, immunosuppression due to congenital or acquired immune deficiency, whether due to concurrent disease (e.g. AIDS, Lymphoma, Leukemia), concomitant cancer therapy (cytotoxic drugs, radiation), or immunosuppressive therapy (e.g. corticosteroids, DMARDs), symptomatic urinary tract infection, febrile illness, patients requiring chronic treatment with certain antibiotics that may interfere with the effectiveness of BCG, any previous allergies or severe reactions to BCG, 6) patients with contraindications to acupuncture/electrostimulation including pregnancy or patient who are trying to become pregnant, patients with a pacemaker, thrombocytopenia, neutropenia, receipt of acupuncture 3 months prior to enrollment, 7) patients requiring chronic treatment with certain antibiotics (e.g. fluoroquinolone therapy, antimycobacterial therapy) that may interfere with the effectiveness of BCG. Fluoroquinolone therapy may decrease the efficacy of intravesical BCG. Antibiotic therapy for ongoing treatment of active tuberculosis will decrease the efficacy of intravesical BCG, and 8) uncontrolled or concurrent illness including, but not limited to, ongoing or active infection, symptomatic congestive heart failure, unstable angina pectoris, cardiac arrhythmia, or psychiatric illness/social situations that would limit compliance with study requirements.

### 2.4 Recruitment procedures

A member of the research team will manually review the weekly schedules of full-time uro-oncology faculty (n = 6) and fellows (2) to identify patients who met eligibility criteria as potential study subjects. Patients eligible to participate in the study will be approached by the primary investigator or the Attending Physician or Advanced Practice Provider in the clinic following their post-operative visit during which pathology was discussed and they were counseled regarding BCG induction therapy. Study details are provided to the patient and informed consent will be discussed at this time. If informed consent is granted by the patient, the patient is entered into this trial.

### 2.5 Data collection

Baseline data collection will include demographics, clinical characteristics, bladder cancer symptoms, quality of life (QOL), and medication use. Prior to initiation of induction BCG, baseline data will be collected at the time of randomization, or at a subsequent baseline study visit or via the phone, e-mail or mailing, depending on patient preference.

Baseline data collection will include 1) clinicopathologic and demographic data; 2) a patient-reported voiding and symptoms assessment using the EORTC QLQ-NMIBC-24 [14,18] Survey, a validated quality of life questionnaire assessing the domains of urinary symptoms including OAB symptoms, malaise, intravesical treatment issues, future worries, bloating/flatulence, sexual function/intimacy, concerns regarding risk to partners related to therapies designed specifically for patients with NMIBC; 3) patient-reported quality of life as assessed via the EORTC-QLQ-C30 Survey [15,19,20], a validated generic quality of life instrument assessing the domains of depression, anxiety, physical function, pain interference, fatigue, sleep disturbance, and ability to participate in social roles and activities ranked on a 4-point Likert scale with an additional 7-point rating scale for overall health and quality of life; 4) current medication use; and 5) current use of complementary, alternative and integrative therapies (Appendix 1); 6) acupuncture expectancy and treatment preference (Appendix 2).

Follow-up data collection includes feasibility, efficacy, and implementation measures (Table 1) plus healthcare utilization assessments. On a weekly basis, prior to BCG instillation and adjunctive acupuncture therapy (if applicable), patients in both the intervention and attention/waitlist control arms will be assessed weekly using the following metrics: EORTC-QLQ-NMIBC-24, EORTC-QLQ-C30, and Medication Journal. In Week 1 the Assessment of complementary, alternative, and integrative therapies and Acupuncture expectancy and treatment preference will also be assessed. In the follow-up assessment the follow-up-assessment of complementary, alternative, and integrative therapies and exit questionnaire will be administered.

**Table 1:** Outcome measures.

Feasibility Measures	1. *Enrollment*: Proportion of patients successfully recruited compared to the number of eligible patients with whom the trial was discussed. 2. *Retention*: Proportion of patients in the experimental (acupuncture) and attention/waitlist control arms who successfully completed the study procedures 3. *Adherence*: Proportion of patients in the experimental (acupuncture) arm who completed the treatment protocol.
Efficacy Measures	1. *BCG Adherence*: a. Completion of BCG: Number of cycles (1–6) completed of planned six induction treatments (yes/no) b. Delays in BCG administration/Treatment breaks required. If so: length of delay (weeks) and reason for delay (irritative bladder symptoms, Infection, logistics/scheduling issues) will be captured c. Dose reductions in BCG: If so, changes in dose (e.g., 50%, 33%, 25%) are recorded as is the reason for the dose reduction, and the cycle at which dosing was changed. 2. *Patient-reported outcomes*: a. EORTC QLQ-C30 b. EORTC QLQ-NMIBC-24) 3. Medication Journal detailing adjunctive medication and complementary therapy utilization each week for the management of treatment-related symptoms 4. Healthcare Utilization a. Number of patient phone calls/eCare messages regarding intravesical therapy-related complaints b. Number of unplanned presentations to the Urology clinic, Emergency room, Urgent Care, Primary Care/Family Medicine Practice for intravesical therapy-related complaints/concerns 5. Intravesical BCG Dwell time, e.g., how long the intravesical BCG was able to be held by the patient prior to voiding each week 6. Weekly assessment of Severe Adverse Events related to Acupuncture (CTCAE v.5.) 7. Weekly survey of patients undergoing treatment in the acupuncture arm regarding acupuncture-related side effects
Implementation Measures	1. Qualitative summary assessment of the impact of the trial on clinic workflow, amount of time spent by patients undergoing acupuncture treatment 2. Amount of time patients spent in the clinic each visit date.

Weekly healthcare utilization will be measured by the number of patient phone calls and eCare messages regarding intravesical-therapy related complaints and unplanned presentations to the emergency room, clinic, and their local providers. BCG Treatment adherence to each successive BCG therapy will be measured via receipt of complete induction course (yes/no), need for delay of therapy (if yes, by how long), need for decrease in BCG-dose, timing of need for decrease in BCG-dose. Intravesical therapy dwell time will be measured by time from BCG instillation to time of first void. Patients will be requested to either text or call the research coordinator for the study upon void. If the research coordinator has not heard from the patient within 4–5 h of their clinical appointment, they will contact the patient by phone call, text, or email to inquire as to what time the patient voided following BCG instillation. Adverse events will be recorded on a weekly basis using the CTCAE v5. Patients in the acupuncture arm will be surveyed weekly prior to acupuncture regarding side effects and potential adverse events associated with the intervention using a standardized symptom assessment form (Appendix 3).

Intervention implementation metrics will be assessed via the following:

• Burden to physicians/healthcare providers will be assessed via survey to the nursing staff, nursing managers and clinic manager regarding the burden of the acupuncture procedure to clinic workflow, delays in providing care, and overall assessments of trial implementation success.
• Timing of visits will be assessed via recording of time of presentation, time of initiation of therapy, and time leaving the clinic.

### 2.6 Randomization

Patients who provide consent are randomized 2:1 to receive weekly acupuncture pre-BCG treatment plus standard of care symptom management (Arm A) or to the attention/waitlist control arm (Arm B), standard of care symptom management plus four coupons for acupuncture after study completion.

### 2.7 Standard of care procedures

Patients undergoing induction BCG will present weekly to clinic, 1 h prior to their scheduled intravesical instillation, at which time a urinalysis will be obtained to rule out gross hematuria or concern for urinary tract infection.

Patients will then be brought into the clinic exam room where a foley catheter will be placed by a nurse or advanced practice provider. The bladder will be emptied completely. Then the TICE BCG solution (50 mL) will be instilled into the catheter over 1–3 min by gravity. The catheter will be removed, and the patient will be discharged from clinic. As per routine practice, patients will be instructed to attempt to maintain the medication in their bladder (avoid voiding) for 2 h, at which time they may void the medication normally with appropriate precautions to ensure deactivation of the live attenuated vaccines. This procedure is repeated weekly for six weeks. If patients report gross blood in the urine or are found to have a urinalysis consistent with urinary tract infection at the time presentation or are having severe symptoms of cystitis or bladder pain, treatment is deferred for that week in consultation with their treating physician.

*Symptom management*. All patients will receive standard of care medications to treat symptoms (e.g., anticholinergics such as oxybutynin, tolterodine, darifenacin, solifenacin, trospium, fesoterodine; Beta-3 adrenergic agonists such as mirabegron, local analgesics such as phenazopyridine, narcotic medications such as oxycodone if necessary, nonnarcotic medications such as Tylenol and non-steroidal anti-inflammatory medications) as indicated.

## 3 Acupuncture procedures

### 3.1 Acupuncture protocols: acupuncture and attention/waitlist control acupuncture

#### 3.1.1 Arm A: acupuncture intervention

After providing a urinary sample for a screening urinalysis prior to BCG treatment, patients randomized to the acupuncture intervention will be escorted to a consultation room where they will meet with the study acupuncturist. Over the next 45–60 min the following protocol will be implemented:

*Questionnaires*: The patient will be asked complete the EORTC-QLQ-C30 and EORTC-QLQ-NMIBC questionnaires, Medication Journal, and patient-reported assessment of acupuncture-related sided effects.

*Position*: The patient will lay in a comfortable position on a portable acupuncture table. Specifically, the patient will lay in the lateral recumbent position on their preferred side.

**Fig. 2:**
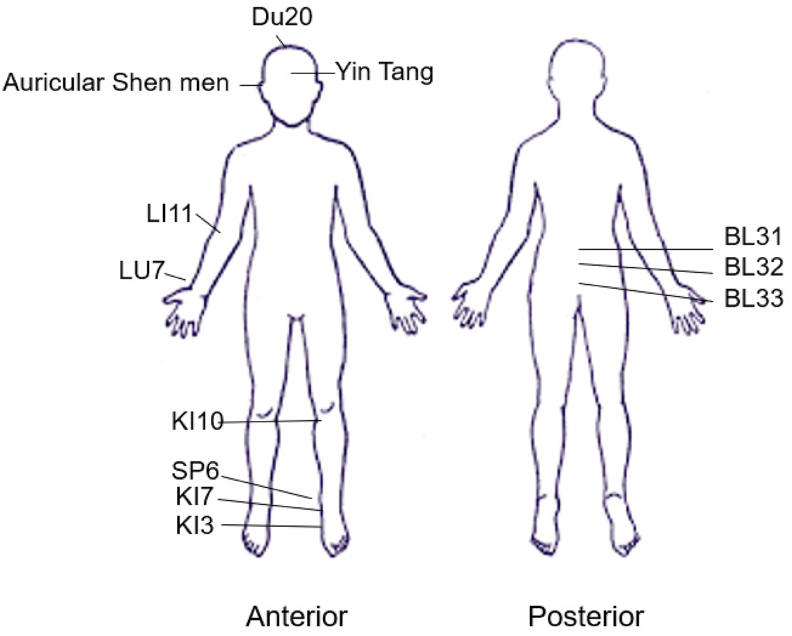
Acupuncture point protocol.

*Acupoints*: Standard techniques for point location [21] will be utilized and Clean Needle Technique [22] will be followed. See Fig. 2 for acupoint locations. The acupuncture points will be needled unilaterally at KI3, KI7, KI10, and SP6 on the lower leg. On the contralateral sided LI11 and LU7 will be needled on the upper extremity. The points BL31, BL32, and BL33 will be needled bilaterally near the sacrum. The points Yin Tang (EX-HN3) and DU20 will be needled on the midline and auricular Shen Men will be needled unilaterally on the same side as the upper limb points. There will be a total of 15 needle sites. Electro-stimulation will be administered to SP6 and KI7 at 1–10 mA with a frequency of 20 Hz. Needles will be retained for 30 min. Specific acupoint selection is based on previously published studies [[6], [7], [8]] indications according to Traditional Chinese Medicine (TCM) theory [21] and current understanding of innervation and neuromodulation of acupoints [12,21]. After being retained for 30 min the needles will be removed and the patient will be moved to the treatment room where BCG will be administered according to standard protocol.

*Acupuncture devices*. 32-40-gauge x 30 mm–40mm Seirin acupuncture needles will be used on all acupoints. The needles and guide tubes are individually packaged and sterile. The manufacturer of Seirin acupuncture needles conforms to with GMP, AAMI (American National), ISO 9002, CE Mark (European Common Market), and WHO (World Health Organization) guidelines for quality and safety [23]. Electroacupuncture (EA) machines used will be FDA compliant medical devices [24].

*Licensing and credentialing of acupuncturists*: Acupuncture treatments will be performed by Washington State licensed acupuncturists with National Certification Commission for Acupuncture and Oriental Medicine (NCCAOM) certification. Study acupuncturists will be credentialed by the Fred Hutchinson Cancer Center (FHCC) and UWMC to provide acupuncture to oncology patients and will be supervised by the FHCC Acupuncturist.

#### 3.1.2 Arm B: attention/waitlist control arm

To minimize differences in experience between the acupuncture intervention and attention/waitlist control arms, patients in the attention/waitlist control arm will be escorted to the same Consultation room where the acupuncture is performed and asked to lie in a comfortable position (similar to Arm A) prior to their intravesical therapy for an identical period of time before being moved to a treatment room for intravesical instillation of BCG, according to the standard protocol detailed above. At the conclusion of the trial participants in the attention/waitlist control arm will receive coupons to receive four free acupuncture appointments. All patients will receive standard of care to treat symptoms (e.g., narcotic and nonnarcotic medications, local analgesics, anticholinergic medications) as indicated.

#### 3.1.3 Acupuncture protocol development

The acupuncture study intervention was developed by a team of acupuncture experts (SV, JS) and is based on previous studies of acupuncture for bladder-related symptoms [[6], [7], [8], [9],^12^]. The acupuncturists had treated oncology patients for at least fifteen years. The study acupuncture point protocol was derived by consensus with respect to Traditional Chinese Medicine differential diagnosis, treatment principles, and point prescriptions (see Table 2).

**Table 2:** Acupuncture point protocol.

Full body acupuncture points
Bilateral points
• BL31
• BL32
BL33
Unilateral lower extremity points
• KI10
• SP6
• KI7
• KI3
Contralateral upper extremity points
• LI11
LU7
Midline points
• GV20
• Yin Tang (EX-HN3)
Unilateral auricular acupuncture points
• Shen Men
**Electroacupuncture points (1**–**10 mA at 20Hz)**
Unilateral points
• SP6
• KI7

#### 3.1.4 Acupuncture protocol

The acupuncture treatments consist of six sessions lasting 60 min each (including a 30-min needle retention time) that are administered over six weeks (i.e., 1 session per week). Acupuncture treatment is standardized. Table 2 displays the acupuncture point protocol. Specific acupoint prescription is based on previously published studies [[6], [7], [8]], indications according to Traditional Chinese Medicine (TCM) theory [21] and current understanding of innervation and neuromodulation of acupoints [9,12]. The study acupuncturists will utilize an even needle technique to elicit the de qi sensation. Electrical stimulation will be applied. Standard techniques for point location, insertion depths and angles [21] and Clean Needle Technique [22] will be followed. Acupuncturists will be instructed to achieve the de qi sensation in which patients experience a dull or achy feeling; this sensation is indicative of effective needling [25]. Stainless steel, single-use, sterile disposable needles will be used for the intervention. Study needles and guide tubes are individually packaged and sterile. The acupuncture needles are 32-40-gauge x 30 mm–40mm Seirin acupuncture needles. The manufacturer of Seirin acupuncture needles conforms with GMP, AAMI (American National), ISO 9002, CE Mark (European Common Market), and WHO (World Health Organization) guidelines for quality and safety [23]. FDA compliant Electrostimulation (EA) machines will be used [24]. The needles will be retained for 30 min, the needles are then removed, and the patient is moved to the treatment room where BCG will be administered according to standard protocol.

#### 3.1.5 Attention/waitlist control acupuncture

At the end of the study, participants randomized to the attention/waitlist control group will receive coupons to receive four free acupuncture treatments in the UWMC Uro-oncology Clinic. Attention/waitlist control acupuncture treatments will be conducted as clinical acupuncture visits including TCM review of systems, assessments of chief complaints, and TCM diagnoses. Acupoint treatment plans will be based on chief complaint and TCM diagnosis for each acupuncture visit and collected in REDCap. Adverse events related to the acupuncture procedures will be collected.

## 4 Acupuncturist training and credentialing

### 4.1 Credentialling acupuncturists

True and attention/waitlist control acupuncture interventions will be performed by Washington State licensed acupuncturists with National Certification Commission for Acupuncture and Oriental Medicine (NCCAOM) certification. Study acupuncturists are hired by Fred Hutchinson Cancer Center and credentialed by the Fred Hutchinson Cancer Center (FHCC) and University of Washington Medicine to provide acupuncture to oncology patients and are supervised by the FHCC Acupuncturist.

### 4.2 Human subjects training

All acupuncturists and study staff completed human subjects training per institutional guidelines for conducting human research.

### 4.3 Virtual training

Live virtual training sessions for study acupuncturists were developed, conducted and lead by the FHCC Acupuncturist. These training sessions included an introduction, which covered general information about the study, acupuncture procedure training, Epic training specific to the study, and REDCap project training. The Introduction training materials included information on the purpose of the study, information on the study site, the study design and objectives, the basic eligibility criteria, patient confidentiality, basic adverse event assessments and reporting, study contact information, documentation of patient treatments, acupoint protocol diagrams, and patient scheduling.

### 4.4 Training manuals

Written training manuals for acupuncturists were developed and distributed to each acupuncturist. They cover the same information as in the virtual training. The manuals will be updated throughout the duration of the trial, as needed.

### 4.5 In-person acupuncturist training

All study acupuncturist attended an on-site orientation and an acupuncture protocol training session. The on-site orientation included a study overview, tour of the clinic and locations of supplies, and introductions to study research coordinators and nursing staff. This orientation included site-specific administrative and infectious disease protocols as well as badging, parking and transportation logistics. During the acupuncture protocol training session, each study acupuncturists administered the acupuncture procedure on a test subject and was trained on needling methods and techniques for standardization across all acupuncture treatments. The study acupuncturists preformed a practical demonstration of study procedures with regard to greeting and assessing the study patient; clean needle technique and hand hygiene; and the acupoint location and needling technique for the protocol (Table 3). Ongoing monthly virtual meetings with study acupuncturists led by the FHCC Acupuncturist were also conducted to address questions and concerns of the study personnel.

**Table 3:** Training and quality assessment.

Greeting
• Neutral demeanor
• Standardized greetings
• Inquiry about adverse events
• Standardized talking points
**Set up**
• Clean field technique
• Hand hygiene
• Patient positioning
• Point swabbing
**Point location**
• Competency demonstration
**Technique**
• Insertion
• Stimulation
• Estim
• Obtaining de qi
• Needle removal

### 4.6 Randomization and blinding of acupuncture status

Once a patient is enrolled in the trial, they are randomized by the research coordinator by 2:1 (Active:Attention/Waitlist Control) and assigned using a random permuted block design.

## 5 Data collection by acupuncturists

Study acupuncturists will indicate whether study patients receive all acupoints or fewer acupuncture points than the full study protocol for any reason. Acupuncturists will also monitor adverse events related to the study procedures by checking whether the patient report any pain, bruising, bleeding, or other reactions during the acupuncture session and since the previous acupuncture session. Data will be collected in REDCap at the time of the acupuncture treatment.

Study acupuncturists will collect TCM diagnostic data including TCM diagnoses for attention/waitlist control acupuncture treatments. Acupoint treatment plans will be based on chief complaint and TCM diagnosis for each acupuncture visit and collected in REDCap. Acupuncturists also monitor protocol adverse events related to the acupuncture procedures by checking whether the patient report any pain, bruising, bleeding, or other reactions during the acupuncture session and since the previous acupuncture session.

### 5.1 Primary endpoints

The primary endpoints of this study were developed to evaluate the feasibility of performing acupuncture prior to weekly BCG therapy for NMIBC and to measure the effect of acupuncture on BCG adherence (Table 1). As such, primary endpoints for this study include: 1) evaluation of BCG protocol experience, 2) patient-reported outcomes of overall quality of life and urinary symptoms, and 3) quantification of medication usage for symptom management. These endpoints will be measured by comparing rates of: 1) BCG instillation adherence (out of a possible planned six treatments), 2) treatment interruptions, 3) weeks missed, 4) intravesical BCG dwell times (as measured in minutes), and 5) requirement for dose reductions between the acupuncture and control arms, 6) responses of patients in the experimental and control arms to the EORTC-QLQ-NMIBC-24 symptom index, which was specifically designed to assess bladder and bowel symptoms for patients with NMIBC including assessments of impact of intravesical therapy, and the EORTC-QLQ-C30, which assesses general quality of life, 7) median weekly pill counts of adjunctive medications for BCG-related symptoms standardized by dosage across medication types. The pill diary will include topical analgesics such as phenazopyridine, anticholinergic medications, Beta-3 adrenergic receptor agonists (e.g., mirabegron), systemic pain medications including acetaminophen, nonsteroidal anti-inflammatory agents, narcotics, and other naturopathic therapies targeted at symptoms of dysuria, bladders spasms, and pelvic pain. These assessments will be compared between patients by week of treatment (from week 1 to week 6).

## 6 Results and discussion

### 6.1 Data analytic plan

Feasibility objectives will be described using qualitative report. Outcomes includes: (1) Trial recruitment (proportion enrolled versus eligible, reason for not enrolling) and retention (proportion dropped out); (2) BCG instillation adherence, defined as completion of at least 5 treatments; (3) Clinic staff's responses to surveys assessing the healthcare burden of this protocol and time spent undergoing the acupuncture therapy; and (4) Adverse events associated with acupuncture therapy. Demographics of patients recruited/enrolled versus not recruited/enrolled will also be compared using Student's t-test and Chi-square test as appropriate.

Secondary efficacy objectives comparing the acupuncture treatment and attention/waitlist control arms will utilize Student's t-test, Wilcoxon Rank Sum test and Chi-square test as appropriate for parametric, non-parametric and categorical variables. These outcomes include: (1) BCG instillation adherence, defined as completion of at least 5 treatments; (2) BCG treatment interruptions, defined as any versus none and weeks missed (measured as any vs none, and total weeks); (3) Intravesical BCG dwell times (measured in minutes); (4) Requirement for dose reductions (measured as yes/no); (5) Patients' responses to the EORTC-QLQ-NMIBC-24 symptom index and EORTC-QLQ-C30 at all time points; and (6) Median weekly pill counts standardized by dosage across medication types.

### 6.2 Anticipated results

Methods were developed to train acupuncturists and staff to implement a randomized attention/waitlist-controlled clinical trial to test the effects of a standardized acupuncture protocol on BCG-related symptoms. We hypothesize that the study is feasible and can be integrated into the clinical workflow of the UWMC Urology clinic with relatively minimal disturbance of the current procedures given that we will be using a consultation room which is currently not utilized for clinical visits during the day. Additionally, given that patients are requested to be in clinic 1 h prior to their BCG instillation to complete standard of care urinanalysis to evaluate for contraindications to proceeding with BCG due to infection or hematuria, we do not anticipate that this study will place further undue time burden upon patients. We do not anticipate problems with study recruitment and retainment in this population and that these patients will have a high level of protocol adherence.

We hypothesize that patients in the experimental group will have a higher proportion of completing the standard 6-weekly BCG regimen with less interruptions and fewer required dose reductions of BCG. We also hypothesize that patients in the experimental group will report similar general quality of life and NMIBC-specific symptoms initially, however, as patients advance in their treatments from 1 to 6 weeks, we anticipate that patients in the experimental arm will endorse improved quality of life and lower levels of bladder/bowel symptoms compared to the control arm. Furthermore, we predict that patients randomized to the experimental arm will have higher rates of protocol retention, treatment adherence, and median intravesical dwell times of the BCG at each treatment; that patients in the experimental arm will report taking lower amounts of adjunctive medications for bladder-relates symptoms; and that the discrepancy in adjunctive treatments taken will increase across the 6 weeks of therapy as the BCG-related symptoms are expected to increase with each subsequent intravesical dose of BCG.

We hypothesize that the acupuncture procedures in the experimental arm will be well tolerated without clinically meaningful adverse events other than minimal bruising and occasional soreness at the needling site. The trial is underway and trial results are anticipated in 2022.

### 6.3 Limitations

Anticipated limitations of this planned pilot trial include the small sample size which may limit the power to detect clinically significant differences in key outcome parameters. However, the data generated as well as clinical workflows developed to permit trial completion will inform the design of a larger study that will have the capability of evaluating efficacy of acupuncture to reduce intravesical BCG-related sides effects and increase tolerability.

## Funding

Early Phase Clinical Research Support Application, Cancer Center Support Grant.

## Declaration of competing interest

The authors declare that they have no known competing financial interests or personal relationships that could have appeared to influence the work reported in this paper.

## Figures and Tables

**Figure:**
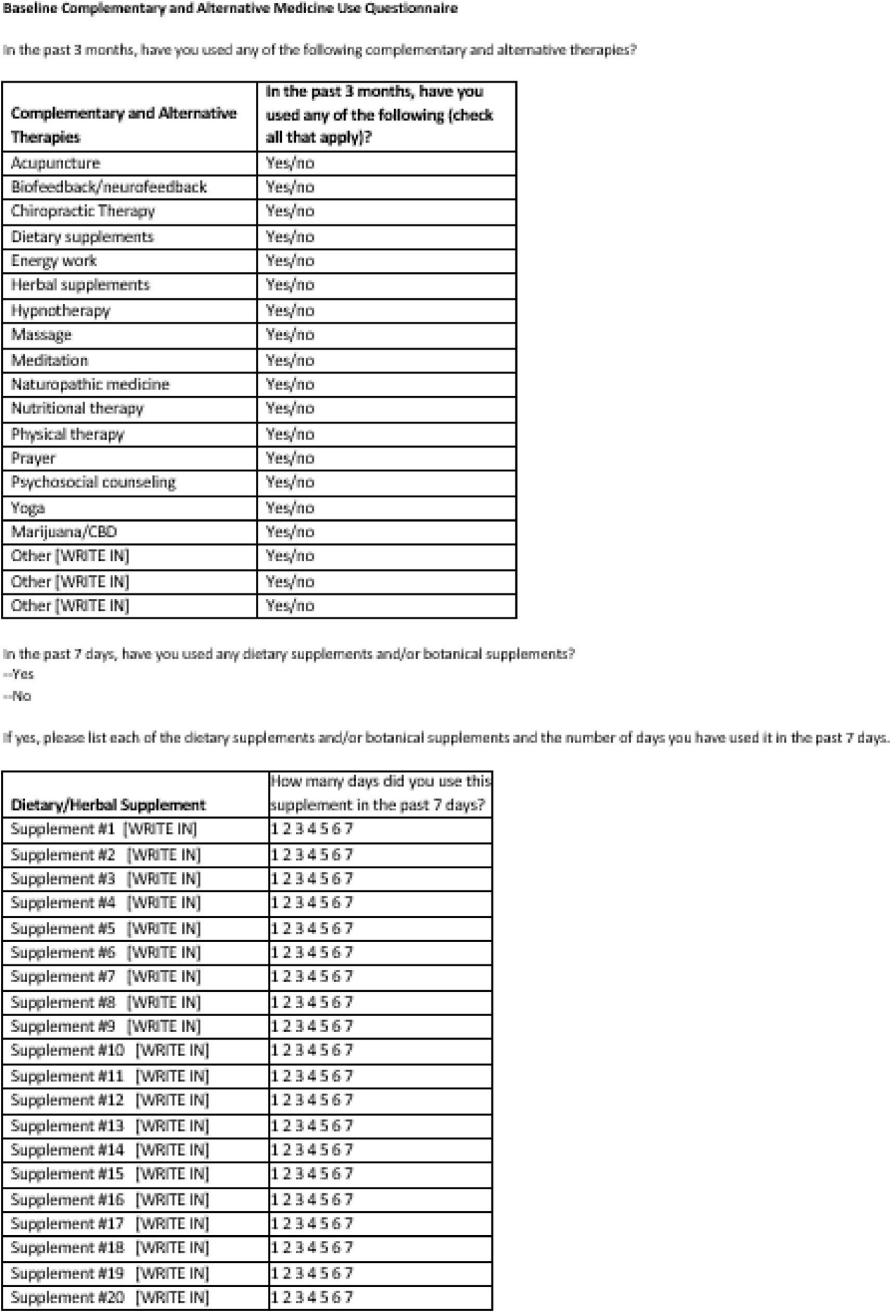


**Figure:**
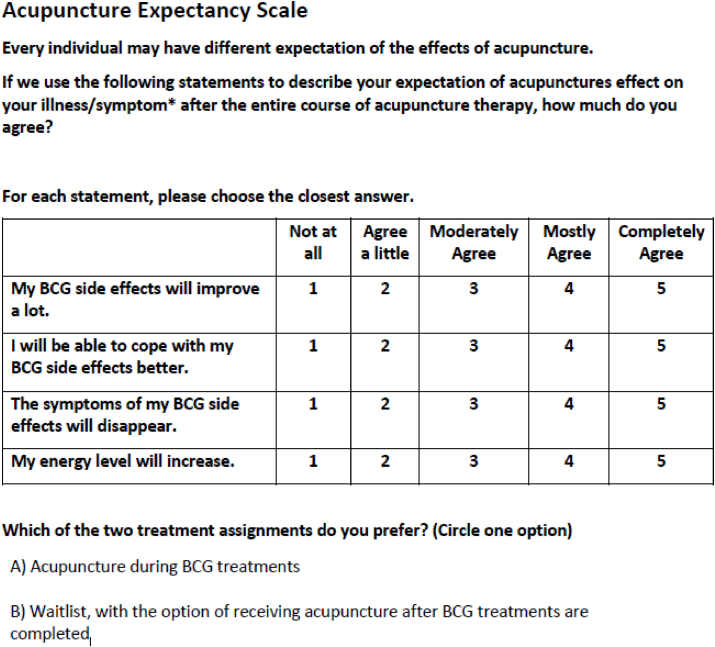


**Figure:**
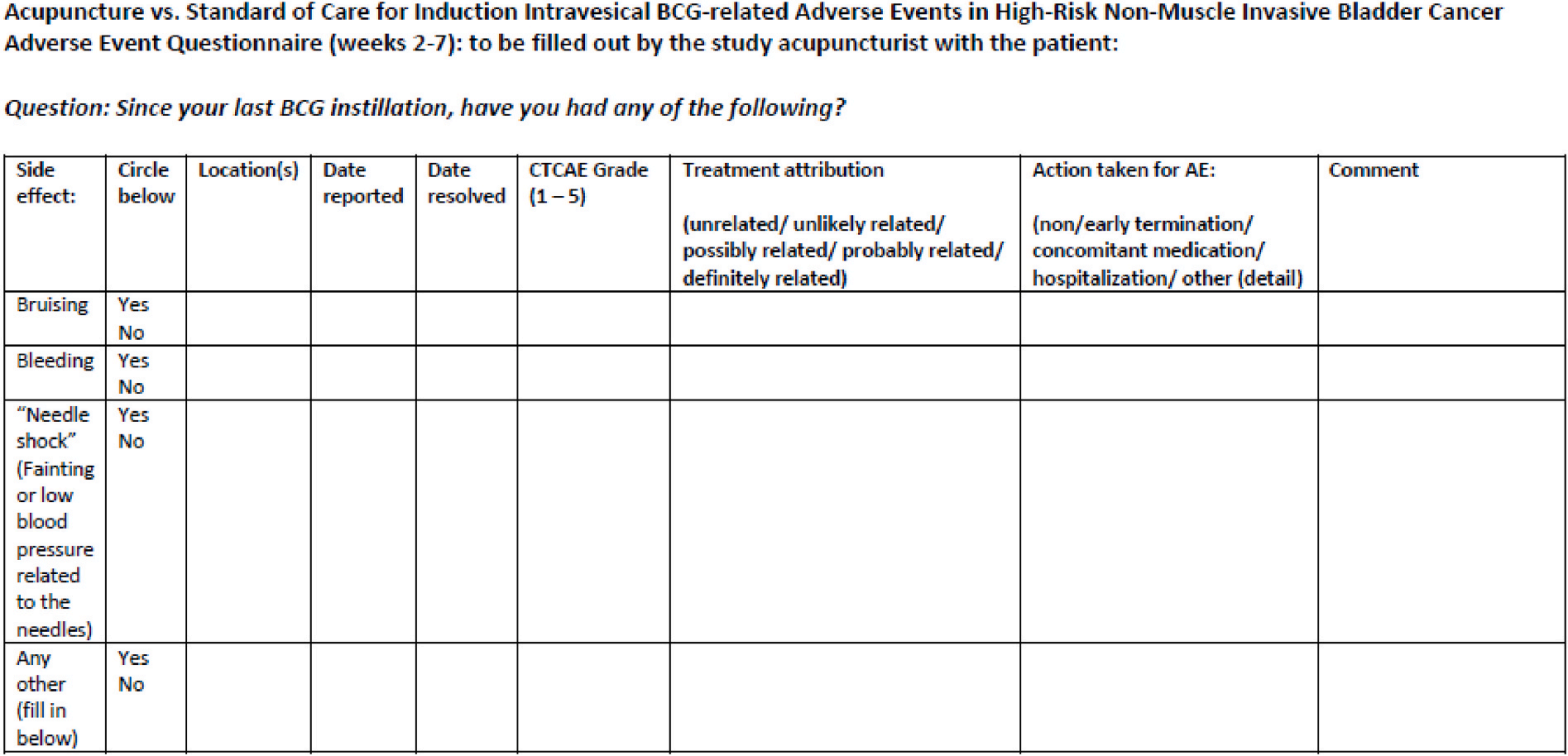


## Data Availability

No data was used for the research described in the article.
